# Return of the Number of Recruits for the Army, Inspected at Glasgow, from the 1st of January, 1817, to the 20th of June, 1823, Divided into Annual Periods, Wherein the Number Deemed Fit for the Service Are Distinguished from Those Considered to Be Unfit; with a Specification of the Causes of Rejection

**Published:** 1824-08

**Authors:** 


**Art. II.**—*Return of the Number of Recruits for the Army, inspected at Glasgow, from the 1st of January,* 1817, *to the 20th of June,* 1823, *divided into annual Periods, wherein the Number deemed fit for the Service are distinguished from those considered to be unfit; with a Specification of the Causes of Rejection.*

| | 1817 | 1818 | 1819 | 1820 | 1821 | 1822 | To June 1823 | Total |
|---|---|---|---|---|---|---|---|---|
| Deemed fit for the service | 506 | 460 | 625 | 895 | 574 | 807 | 520 | 4387 |
| Deemed unfit for the service | 107 | 133 | 180 | 243 | 214 | 315 | 176 | 1368 |
| Total number inspected | 613 | 593 | 805 | 1138 | 788 | 1122 | 696 | 5755 |
| Marks of punishment | 0 | 2 | 7 | 7 | 10 | 7 | 1 | 34 |
| General debility, or unhealthy appearance | 18 | 18 | 11 | 11 | 0 | 32 | 11 | 101 |
| Cutaneous diseases | 1 | 2 | 3 | 12 | 0 | 5 | 6 | 29 |
| Cicatrices of ulcers | 8 | 2 | 1 | 1 | 9 | 8 | 4 | 33 |
| Scrofula, or scrofulous appearances | 8 | 6 | 14 | 10 | 17 | 17 | 6 | 78 |
| Diseases of the eyes, or defect of vision | 8 | 10 | 3 | 4 | 12 | 23 | 22 | 82 |
| Deafness | 0 | 2 | 1 | 0 | 1 | 2 | 1 | 7 |
| Diseased teeth, including the loss of teeth | 0 | 0 | 1 | 0 | 1 | 0 | 1 | 3 |
| Polypus | 0 | 0 | 0 | 0 | 0 | 4 | 2 | 6 |
| Ill-formed chest, with either depressed or protruding sternum | 5 | 4 | 20 | 14 | 26 | 24 | 15 | 108 |
| Malformation of the spine | 0 | 2 | 0 | 1 | 4 | 1 | 2 | 10 |
| Fractures, dislocations, tumors, or malformations of either the superior or inferior extremity | 27 | 42 | 56 | 58 | 52 | 58 | 37 | 330 |
| Inguinal hernia, or disposition to hernia of both rings | 0 | 0 | 0 | 0 | 0 | 3 | 1 | 4 |
| ——— of right side | 0 | 0 | 2 | 5 | 0 | 8 | 3 | 18 |
| ——— of left side | 1 | 0 | 0 | 2 | 6 | 2 | 0 | 11 |
| Varicose Veins of either legs, hams, or thighs, throughout the course of the vena, saphena, on both sides | 1 | 4 | 13 | 16 | 2 | 3 | 3 | 42 |
| Varicose Veins Do. do. right leg, &c. | 6 | 5 | 14 | 25 | 13 | 18 | 9 | 90 |
| Varicose Veins Do. do. left do. | 10 | 13 | 18 | 18 | 19 | 22 | 17 | 117 |
| Varicose Veins of the spermatic processes of both sides | 0 | 0 | 0 | 2 | 0 | 3 | 1 | 6 |
| Varicose Veins Do. do. right do. | 0 | 0 | 1 | 1 | 1 | 0 | 0 | 3 |
| Varicose Veins Do. do. left do. | 0 | 0 | 5 | 40 | 13 | 20 | 11 | 89 |
| Ventral hernia | 0 | 0 | 0 | 0 | 1 | 0 | 0 | 1 |
| Enlargement of both rings | 0 | 0 | 0 | 1 | 1 | 0 | 0 | 2 |
| Do. right abdominal ring | 0 | 0 | 2 | 6 | 0 | 0 | 2 | 10 |
| Do. left do. | 0 | 0 | 0 | 0 | 1 | 0 | 0 | 1 |
| Diseased right testicle, either producing diminution or enlargement | 1 | 2 | 0 | 2 | 0 | 4 | 1 | 10 |
| Do. left do. do. | 4 | 5 | 1 | 1 | 0 | 10 | 1 | 22 |
| Enlarged gland of the groin, and unconnected with scrofula | 1 | 7 | 1 | 0 | 7 | 5 | 6 | 27 |
| Hydrocele | 0 | 0 | 0 | 0 | 2 | 4 | 0 | 6 |
| In-knee'd | 0 | 0 | 0 | 1 | 9 | 4 | 8 | 22 |
| Venereal disease | 0 | 3 | 0 | 0 | 2 | 9 | 2 | 16 |
| | 99 | 129 | 174 | 238 | 209 | 296 | 173 | 1318 |

| | 1817 | 1818 | 1819 | 1820 | 1821 | 1822 | 1823 | |
|---|---|---|---|---|---|---|---|---|
| Brought forward | 99 | 129 | 174 | 238 | 209 | 296 | 173 | 1318 |
| Tenia capitas | 2 | 3 | 0 | 0 | 1 | 3 | 0 | 9 |
| Fractured clavicle | 0 | 0 | 1 | 0 | 1 | 0 | 0 | 2 |
| Defect of speech | 0 | 0 | 1 | 3 | 1 | 1 | 0 | 6 |
| Loss of teeth | 0 | 0 | 1 | 0 | 1 | 0 | 1 | 3 |
| Imperforate urethra, and malformation in the orifice | 0 | 0 | 0 | 0 | 0 | 2 | 0 | 2 |
| An organic disease of the heart | 1 | 0 | 0 | 0 | 0 | 0 | 0 | 1 |
| Asthmatic | 2 | 0 | 0 | 0 | 0 | 0 | 0 | 2 |
| Bubonocele | 1 | 0 | 0 | 0 | 0 | 0 | 0 | 1 |
| Hare-lip | 1 | 0 | 0 | 0 | 0 | 0 | 0 | 1 |
| Fractured patella | 1 | 1 | 0 | 0 | 0 | 2 | 0 | 4 |
| Deformity of the face | 0 | 0 | 1 | 0 | 0 | 0 | 0 | 1 |
| Unable to march | 0 | 0 | 1 | 0 | 0 | 0 | 0 | 1 |
| Subject to mental derangement | 0 | 0 | 0 | 1 | 0 | 0 | 0 | 1 |
| Aneurismal enlargement of the carotid artery | 0 | 0 | 0 | 1 | 0 | 0 | 0 | 1 |
| Injuries of the head | 0 | 0 | 0 | 0 | 1 | 5 | 0 | 6 |
| Enlarged bursæ mucosæ | 0 | 0 | 0 | 0 | 0 | 1 | 0 | 1 |
| Ulcers on the trunk | 0 | 0 | 0 | 0 | 0 | 1 | 0 | 1 |
| Excrescences of the eyelids | 0 | 0 | 0 | 0 | 0 | 1 | 0 | 1 |
| Diseased viscera | 0 | 0 | 0 | 0 | 0 | 1 | 0 | 1 |
| Diseased bones | 0 | 0 | 0 | 0 | 0 | 1 | 0 | 1 |
| Ulcerated throat | 0 | 0 | 0 | 0 | 0 | 1 | 0 | 1 |
| Injuries of the abdominal muscles, and diseased appearances of the abdomen | 0 | 0 | 1 | 0 | 0 | 1 | 1 | 3 |
| Total | 107 | 133 | 180 | 243 | 214 | 315 | 176 | 1368 |

*Remarks.*

The accompanying Return I was, in the first instance, induced to compile merely from motives of curiosity as to the number of men enlisted during the last seven years in this district alone, as also the number of men rejected since, which would afford an exact total of the men that entered the service; but, on looking over the diseases for which these men were rejected, I was struck with some peculiarities amongst them, which I have never seen brought to public notice; neither had I a prior opportunity of observing. This influenced me to form a class of the different causes of rejection, which I have put under as few heads as possible; but regret, in so doing, that it escaped my notice, in the annexed Return, to pay more attention to the nosological arrangement at present established for the use of the army.

In making out this list of diseases for which so many men have been rejected, there has been some difficulty in placing them in so small a compass, owing to some unfixed rule in the registry: for instance, *unable to march* appears in the return, the registry affording no cause as to the inability; whereas, if it originated in flat feet, &c., it might have been included under the heads of Diseases of the Extremities, &c. With regard to diseased testicles, varicose veins of the legs and spermatic processes, the want of discrimination as to which side the disease was to be ascribed, caused some confusion: however, where doubt has arisen, I have divided them to either extremity.

By the Return, the number of men rejected for varicose veins makes a prominent part of the total; but the singularity exists in the frequency of this disease in the left lower extremity to that of the right: in the vein saphina alone it is as 117 to 90, even allowing an equal number to each leg of these cases, where, as I before mentioned, any doubts existed in the registry. This difference is extremely striking in the spermatic processes, where the disproportion is in favour of the left in the ratio of 89 to 3. This is again supported by the frequency of diseased testicles being as 22 to 10; and which may be accounted for, in my opinion, as being produced by the same cause as that producing the varix in the vessels returning the blood from the testicle into the circulating fluid.

The frequency of enlargement of the right abdominal ring, and, of course, the facilitating the descent of the intestine, is another circumstance worthy of remark. The cause, I am aware, is attributed to the greater exertions to which we are, in general, more accustomed to make with the right extremities than the left. This, however, is in contradiction to the general rule, which lays down that the more exertion the living fibres of the muscles are accustomed to, the greater is their power, and, of course, less likelihood of their texture being relaxed.

The causes why varicose veins of the left side are more frequent than that of the right, I conceive anatomy points out to us; for, if we reflect that the inferior cava is principally formed by the junction of the two common iliac veins, and that its situation is upon the right side of the bodies of the vertebræ, and of course more to the right than the descending aorta; and that the left iliac vein has to traverse a much greater extent of pelvis to form a junction with the right, and, in most instances, that the vein has to pass under the right iliac artery for this purpose,—may not the action of such a vessel as the common iliac artery, preventing the return of blood from the lower extremity, be a sufficient cause to produce a distention of the coats of the vessel, and subsequent varix? If so, the veins of the chord are, of course, subject to the same inconvenience, as well as the vena saphena, which, although no doubt effected by any distention of the femoral vein, has, prior to emptying itself into that vessel, to quit the superficial course which it had up the thigh,—namely, between the integuments and sartorius muscle, and penetrate that fascia now known by the name of cribriform fascia, and which has been, in some instances, said to be the seat of stricture in cases of hernia.

